# The influence of renal function on surgical outcomes of vitrectomy in patients with proliferative diabetic retinopathy

**DOI:** 10.3389/fendo.2025.1592618

**Published:** 2025-07-16

**Authors:** Qiongzhen Yuan, Zhouquan Yang, Wei Fan, Xiaofan Chen, Huan Zou, Rongdi Yuan

**Affiliations:** Department of Ophthalmology, Xinqiao Hospital, Army Medical University, Chongqing, China

**Keywords:** diabetes mellitus, proliferative diabetic retinopathy, renal function, surgical outcomes, vitrectomy

## Abstract

**Purpose:**

To investigate the influence of renal function on the surgical outcomes of vitrectomy in patients with proliferative diabetic retinopathy (PDR).

**Methods:**

A secondary analysis was conducted on data from a retrospective cohort study.

**Results:**

A total of 128 eyes with PDR that underwent pars plana vitrectomy (PPV) and were followed up for at least 2 years were enrolled, including 65 eyes in the impaired renal function (IRF) group and 63 eyes in the normal renal function (NRF) group. No significant between-group differences were observed in the proportion of cataract surgery (p = 0.722), intraoperative retinal photocoagulation (p = 0.476), gas tamponade (p = 0.932), silicone oil tamponade (p = 0.254), retinal dialysis and/or iatrogenic retinal breaks (p = 0.447), and 23- or 25-gauge (G) microincision vitrectomy surgery (MIVS) (p = 0.160). Similarly, intergroup comparisons showed no significant differences in the proportion of reoperation (p = 0.883), postoperative vitreous hemorrhage (VH) and/or retinal detachment (RD) (p = 0.919), postoperative neovascular glaucoma (NVG) (p = 0.600), and postoperative diabetic macular edema (DME) (p = 0.794). Notably, the IRF group had worse baseline best corrected visual acuity (BCVA) (p = 0.039) and showed greater BCVA improvement at 3 months (p = 0.008), 6 months (p = 0.047), 1 year (p = 0.007), 2 years (p = 0.003), 3 years (p = 0.009), and 4 years (p = 0.024) after surgery. However, there was no significant difference in postoperative BCVA between the two groups at each follow-up time (all p > 0.05).

**Conclusions:**

Renal insufficiency does not adversely affect the surgical outcomes of PPV in patients with PDR.

## 1 Introduction

Diabetes mellitus (DM) is one of the fastest-growing diseases in the world and is predicted to affect 6.93 billion adults by 2045 (1). Concomitant with the rising DM incidence, its complications, including diabetic retinopathy (DR) and diabetic nephropathy (DN), are increasingly prevalent. DR and DN not only dramatically decrease patients’ life quality but also impose an enormous socioeconomic burden (2–4).

As the most common vascular retinopathy in ophthalmology, DR has become the leading cause of blindness among working-age individuals globally (5). It was predicted that the number of patients with DR would rise to 1.91 billion by 2023 (6, 7). The primary pathophysiology of DR is the long-term effect of hyperglycemia on retinal microvasculature, resulting in increased vascular leakage, retinal ischemia, increased production of vasoactive factors, and finally, neovascularization (8). DR is clinically classified into non-proliferative DR (NPDR) and proliferative DR (PDR) stages. As a more advanced stage of DR, PDR is characterized by neovascularization, during which vision progressively deteriorates due to complications such as vitreous hemorrhage (VH) or retinal detachment (RD), requiring pars plana vitrectomy (PPV) surgery (9, 10).

DN, another severe microvascular complication of DM, affects approximately 40% of patients with DM. It is the primary cause of chronic kidney disease (CKD) and end-stage renal disease requiring dialysis or transplantation in the United States and worldwide (11–13). As both microvascular complications of DM, DN and DR exhibit similar pathophysiological mechanisms and close clinical interrelation (14, 15). Previous studies have demonstrated that hematuria, proteinuria, albumin–creatinine ratio, estimated glomerular filtration rate (eGFR), and glomerulopathy severity were significantly associated with the risk of DR (16, 17). DR severity and diabetic macular edema (DME) were reported to be positively correlated with renal function among southern Chinese patients (18). It was reported that CKD, high urine albumin to creatinine ratio, and low eGFR were associated with DR prevalence among type 2 diabetes mellitus (T2DM) populations (19). Kotlarsky P et al. demonstrated that the degree of renal impairment was directly proportional to the degree of ocular impairment—categorized as normal, mild NPDR, moderate NPDR, or PDR—in patients with T2DM (20). In addition, Fang J et al. revealed positive genetic associations between DR and DN and a significant causal link of DN with both NPDR and PDR (11). However, other studies have demonstrated that DR and DN do not always develop in parallel (21, 22). The relationship between these two microvascular complications may be influenced by obesity, ethnicity, and use of renin–angiotensin–aldosterone antagonists (23).

PPV is a major beneficial surgical treatment for PDR patients with vision-threatening lesions, including preretinal membrane, VH, tractional retinal detachment (TRD), and combined RD. Although most patients can achieve anatomical and visual improvement following PPV, some patients still experience disease progression and vision deterioration. Therefore, it is of great significance to identify factors influencing the surgical outcomes of vitrectomy for PDR. Given the association between DR and DN, renal function may be a vital influencing factor. A retrospective study has demonstrated that severe renal insufficiency may serve as a risk factor for patients with PDR requiring bilateral PPV (24). Additionally, renal dysfunction was reported as the most common cause of death during postoperative follow-up (25). However, limited studies have been conducted on the impact of renal function on PPV surgical outcomes, with a notable absence of long-term follow-up data and a lack of consistent conclusions across existing studies (26–29). In this study, we conducted a secondary analysis of data from a published paper by Nishi et al. (30), which investigated the factors associated with visual outcomes after a long follow-up in patients who underwent vitrectomy for PDR.

## 2 Methods

### 2.1 Study participants and procedures

This study represents a secondary analysis of data derived from a retrospective cohort study conducted by Nishi et al. (30). The original study design was thoroughly described in the primary article by Nishi et al. They retrospectively reviewed the medical records of PDR patients receiving PPV at Yamagata University Hospital between January 2008 and September 2012. They excluded cases with only DME. All patients received three-port 20-gauge (G) or microincision vitrectomy surgery (MIVS) (23-G or 25-G) PPV for persistent VH and TRD. Additionally, all the surgical procedures were performed by two vitreoretinal surgeons. Ultimately, 128 eyes from 100 PDR patients with a follow-up duration of at least 2 years were included. None of the patients received anti-vascular endothelial growth factor (anti-VEGF) therapy as a preoperative adjunct. Pan-retinal photocoagulation was cautiously performed either before or during PPV in all PDR patients. During postoperative follow-up, treatment was performed for progressed cataract, posterior capsular opacification, DME, neovascular glaucoma (NVG), and other vision-threatening lesions. Patients were followed up at 3 months, 6 months, 1 year, 2 years, and 4 years after surgery.

As Nishi et al. (30) stated in the original paper, this study was approved by the Ethics Committee of the Yamagata University Faculty of Medicine (approval number: H26-21) and conformed to the Declaration of Helsinki. Due to the retrospective nature of the study, the institutional review board waived the need for informed consent.

### 2.2 Outcome measures

The following baseline demographic and clinical characteristics were collected: age, gender, duration from awareness of visual loss to surgery, history of hypertension, DM duration, insulin treatment, oral antidiabetic medications, history of DN, history of coronary heart disease and/or stroke, and use of anticoagulant and/or antiplatelet drugs. Systemic factors included systolic blood pressure (SBP), diastolic blood pressure (DBP), heart rate, and blood biochemical parameters, such as preoperative eGFR, glycosylated hemoglobin (HbA1c), creatinine, blood urea nitrogen, uric acid, total cholesterol, triglyceride, and hemoglobin.

The preoperative ophthalmologic parameters included the following: visual acuity, history of intravitreal injection of triamcinolone acetonide, intraocular lens implantation, preoperative retinal photocoagulation, ocular hypertension (>21 mmHg), rubeosis iridis, posterior vitreous detachment, VH, fibrovascular membrane, RD, and macular detachment. The following intraoperative outcomes were included: cataract surgery, silicone oil or gas tamponade, retinal photocoagulation, intraoperative complications (retinal dialysis and iatrogenic retinal breaks), and MIVS application. Lastly, the postoperative outcomes included reoperation, postoperative complications (VH, RD, NVG, and DME), and best corrected visual acuity (BCVA) at each postoperative follow-up time point.

We allocated all patients into two groups according to eGFR: those with normal renal function (NRF) (CKD stage 1–2, eGFR ≥ 60 mL/min/1.73 m^2^) and those with impaired renal function (IRF) (CKD stage 3–4, eGFR < 60 mL/min per 1.73 m^2^) (28, 31). We compared baseline characteristics, intraoperative outcomes (including intraoperative retinal photocoagulation, gas tamponade, and silicone oil tamponade), and postoperative outcomes (reoperation rate, complications, and visual outcomes) between the two groups.

### 2.3 Statistical analyses

Statistical analyses were performed using the SPSS software (version 23.0, IBM Corp., Armonk, NY, USA). For continuous variables with normal distribution (verified by the Kolmogorov–Smirnov test and visual inspection of Q-Q plots), data were expressed as mean ± standard deviation (SD), and group comparisons were performed using an independent samples t-test. Non-normally distributed continuous variables were presented as median [interquartile range (IQR)], and the Mann–Whitney U test was used for two independent groups. Categorical variables were described as numbers and percentages (%) and analyzed using the chi-square test or with Fisher’s exact test when the expected cell counts were less than 5. Differences were regarded to be statistically significant at a two-sided alpha level of p < 0.05.

## 3 Results

### 3.1 Baseline characteristics

The baseline characteristics are shown in **Table 1**. A total of 128 eyes from PDR patients with a follow-up duration of at least 2 years after primary PPV were included in the final analysis, comprising 65 eyes in the IRF group and 63 eyes in the NRF group. Of these, 102 eyes (50 in IRF and 52 in NRF) completed 3-year follow-up, and 91 eyes (45 in IRF and 46 in NRF) completed 4-year follow-up. Compared with the NRF group, the IRF group had a higher percentage of hypertension (81.5% vs. 42.9%, p < 0.001), diabetic nephropathy (92.3% vs. 44.4%, p < 0.001), and anticoagulant and/or antiplatelet agent administration (30.8% vs. 12.7%, p = 0.013) but a lower percentage of rubeosis iridis (6.2% vs. 19.0%, p = 0.027). Additionally, the IRF group had worse baseline BCVA (2.00 logMAR vs. 1.40 logMAR, p = 0.039). For blood biochemical parameters, the IRF group exhibited significantly lower levels of HbA1c (%) (6.80 vs. 7.40, p = 0.026) and hemoglobin (12.20 vs. 13.80, p < 0.001) while showing higher levels of blood urea nitrogen (25.00 vs. 15.00, p < 0.001), creatinine (1.26 vs. 0.69, p < 0.001), uric acid (6.42 vs. 5.11, p < 0.001), total cholesterol (215.15 vs. 195.40, p = 0.019), and triglyceride (156.00 vs. 125.00, p = 0.033). There were no significant differences between the two groups in age, gender, duration from visual loss awareness to the primary surgery, or other baseline variables.

**Table 1:** Baseline characteristics.

Baseline characteristics	IRF group (n = 65)	NRF group (n = 63)	p-Value
Age (years)	56.12 ± 10.94	55.44 ± 11.53	0.733
Gender (male)	49 (75.4%)	42 (66.7%)	0.277
Duration awareness of visual loss to surgery (months)	1.00 (1.00, 4.00)	2.00 (1.00, 5.00)	0.109
Hypertension	53 (81.5%)	27 (42.9)	<0.001
SBP	141.22 ± 21.37	136.48 ± 23.23	0.232
DBP	79.15 ± 12.53	77.81 ± 15.66	0.594
Heart rate (beats/min)	75.89 ± 11.90	73.76 ± 12.73	0.330
DM duration (years)	10.00 (6.50, 13.50)	12.00 (4.00, 18.00)	0.650
HbA1c (%)	6.80 (6.05, 7.65)	7.40 (6.70, 8.30)	0.026
Insulin treatment	38 (58.5%)	38 (60.3%)	0.831
Oral antidiabetic medications	30 (46.2%)	34 (54.0%)	0.377
DN	60 (92.3%)	28 (44.4%)	<0.001
Coronary heart disease and/or stroke	14 (21.5%)	9 (14.3%)	0.285
Anticoagulant and/or antiplatelet drugs	20 (30.8%)	8 (12.7%)	0.013
Intraocular lens implantation	18 (27.7%)	20 (31.7%)	0.616
Preoperative retinal photocoagulation	57 (87.7%)	54 (85.7%)	0.742
Intravitreal injection of triamcinolone acetonide	1 (1.5%)	0 (0.0%)	1.000
Rubeosis iridis	4 (6.2%)	12 (19.0%)	0.027
Ocular hypertension	2 (3.1%)	8 (12.7%)	0.089
Vitreous hemorrhage	54 (83.1%)	48 (76.2%)	0.333
Posterior vitreous detachment	18 (27.7%)	14 (22.2%)	0.475
Fibrovascular membrane	36 (55.4%)	36 (57.1%)	0.841
Retinal detachment	16 (24.6%)	14 (22.2%)	0.749
Macular detachment	10 (15.4%)	8 (12.7%)	0.662
Preoperative BCVA (logMAR)	2.00 (1.00, 2.70)	1.40 (0.70, 2.30)	0.039
HbA1c (%)	6.80 (6.05, 7.65)	7.40 (6.70, 8.30)	0.026
Crea (mg/dL)	1.26 (1.11, 2.44)	0.69 (0.58, 0.86)	<0.001
BUN (mg/dL)	25.00 (17.00, 36.50)	15.00 (13.00, 18.00)	<0.001
UA (mg/dL)	6.42 ± 1.56	5.11 ± 1.38	<0.001
TC (mg/dL)	215.15 ± 49.53	195.40 ± 43.92	0.019
TG (mg/dL)	156.00 (111.00, 203.50)	125.00 (88.00, 190.00)	0.033
Hb (g/dL)	12.20 (10.70, 13.20)	13.80 (12.20, 14.90)	<0.001

IRF, impaired renal function; NRF, normal renal function; SBP, systolic blood pressure; DBP, diastolic blood pressure; DN, diabetic nephropathy; BCVA, best corrected visual acuity; logMAR, logarithmic minimum angle of resolution; HbA1c, glycosylated hemoglobin; Crea, creatinine; BUN, blood urea nitrogen; UA, uric acid; TC, total cholesterol; TG, triglyceride; Hb, hemoglobin.

### 3.2 Intraoperative outcomes

Intraoperative outcomes for the two groups are presented in **Table 2**. No significant group differences were found in the following parameters: the proportion of cataract surgery (50.8% vs. 47.6%, p = 0.722), intraoperative retinal photocoagulation (84.6% vs. 88.9%, p = 0.476), gas tamponade (18.5% vs. 19.0%, p = 0.932), silicone oil tamponade (4.6% vs. 0.0%, p = 0.254), intraoperative complications (retinal dialysis and/or iatrogenic retinal breaks) (13.8% vs. 9.5%, p = 0.447), and MIVS application (38.5% vs. 50.8%, p = 0.160).

**Table 2:** Comparison of intraoperative outcomes between IRF and NRF groups.

Intraoperative outcomes	IRF group (n = 65)	NRF group (n = 63)	p-Value
Cataract surgery	33 (50.8%)	30 (47.6%)	0.722
Intraoperative retinal photocoagulation	55 (84.6%)	56 (88.9%)	0.476
Gas tamponade	12 (18.5%)	12 (19.0%)	0.932
Silicone oil tamponade	3 (4.6%)	0 (0.0%)	0.254
Retinal dialysis and/or iatrogenic retinal breaks	9 (13.8%)	6 (9.5%)	0.447
MIVS	25 (38.5%)	32 (50.8%)	0.160

IRF, impaired renal function; NRF, normal renal function; MIVS, microincision vitrectomy surgery.

### 3.3 Postoperative outcomes

#### 3.3.1 Postoperative complications

There were no significant differences between the two groups in terms of the rates of reoperation (18.5% vs. 17.5%, p = 0.883), postoperative VH and/or RD (24.6% vs. 25.4%, p = 0.919), postoperative NVG (1.5% vs. 5.5%, p = 0.600), and postoperative DME (9.2% vs. 7.9%, p = 0.794) (**Table 3**).

**Table 3:** Comparison of postoperative outcomes between IRF and NRF groups.

Postoperative outcomes	IRF group (n = 65)	NRF group (n = 63)	p-Value
Reoperation	12 (18.5%)	11 (17.5%)	0.883
Postoperative VH and/or RD	16 (24.6%)	16 (25.4%)	0.919
Postoperative NVG Postoperative DME	1 (1.5%) 6 (9.2%)	6 (5.5%) 5 (7.9%)	0.600 0.794

IRF, impaired renal function; NRF, normal renal function; VH, vitreous hemorrhage; RD, retinal detachment; NVG, neovascular glaucoma; DME, diabetic macular edema.

#### 3.3.2 Postoperative visual outcomes

**Table 4** presents the comparison of postoperative BCVA across different follow-up time points. As shown, the two groups demonstrated no significant differences in BCVA at 3 months (p = 0.733), 6 months (p = 0.780), 1 year (p = 0.584), 2 years (p = 0.434), 3 years (p = 0.237), and 4 years (p = 0.234) after PPV. Additionally, the percentage of postoperative BCVA ≥20/40 at 2 years (p = 0.108), ≥20/30 at 2 years (p = 0.614), ≥20/40 at 4 years (p = 0.457), and ≥20/30 at 4 years (p = 0.605) did not differ significantly between groups as well.

**Table 4:** Postoperative BCVA (logMAR) according to renal function category.

Postoperative time points and visual acuity indicators	IRF group (n = 65)	NRF group (n = 63)	p-Value
At 3 months	0.40 (0.15, 1.00)	0.40 (0.05, 1.70)	0.733
At 6 months	0.40 (0.10, 0.85)	0.30 (0.00,1.30)	0.780
At 1 year	0.30 (0.46, 0.76)	0.30 (0.46, 1.15)	0.584
At 2 years	0.30 (0.46, 0.70)	0.30 (0.00, 1.22)	0.434
At 3 years	0.30 (0.00, 0.70)	0.30 (0.01, 1.65)	0.237
At 4 years	0.22 (0.00, 0.67)	0.35 (0.00, 1.82)	0.234
≥20/40 at 2 years	46 (70.8%)	36 (57.1%)	0.108
≥20/30 at 2 years	24 (36.9%)	26 (41.3%)	0.614
≥20/40 at 4 years	26 (57.8%)	23 (50.0%)	0.457
≥20/30 at 4 years	22 (48.9%)	20 (43.5%)	0.605

IRF, impaired renal function; NRF, normal renal function; BCVA, best corrected visual acuity; logMAR, logarithmic minimum angle of resolution.

**Table 5**; **Figure 1** show the comparison of postoperative BCVA (logMAR) improvement between the two groups. The IRF group demonstrated significantly greater BCVA improvement than the NRF group at 3 months (−1.00 logMAR vs. −0.40 logMAR, p = 0.008), 6 months (−1.00 logMAR vs. −0.52 logMAR, p = 0.047), 1 year (−1.22 logMAR vs. −0.60 logMAR, p = 0.007), 2 years (−1.30 logMAR vs. −0.65 logMAR, p = 0.003), 3 years (−1.19 logMAR vs. −0.57 logMAR, p = 0.009), and 4 years (−1.16 logMAR vs. −0.57 logMAR, p = 0.024).

**Table 5:** Comparison of postoperative BCVA (logMAR) improvement.

Postoperative time points	IRF group (n = 65)	NRF group (n = 63)	p-Value
At 3 months	−1.00 (−2.22, −0.08)	−0.40 (−1.30, 0.00)	0.008
At 6 months	−1.00 (−2.30, −0.05)	−0.52 (−1.40, −0.97)	0.047
At 1 year	−1.22 (−2.28, −0.30)	−0.60 (−1.48, 0.00)	0.007
At 2 years	−1.30 (−2.40, −0.30)	−0.65 (−1.48, 0.00)	0.003
At 3 years	−1.19 ± 1.08	−0.57 ± 1.26	0.009
At 4 years	−1.16 ± 1.14	-0.57 ± 1.28	0.024

IRF, impaired renal function; NRF, normal renal function; BCVA, best corrected visual acuity; logMAR, logarithmic minimum angle of resolution.

**Figure 1:**
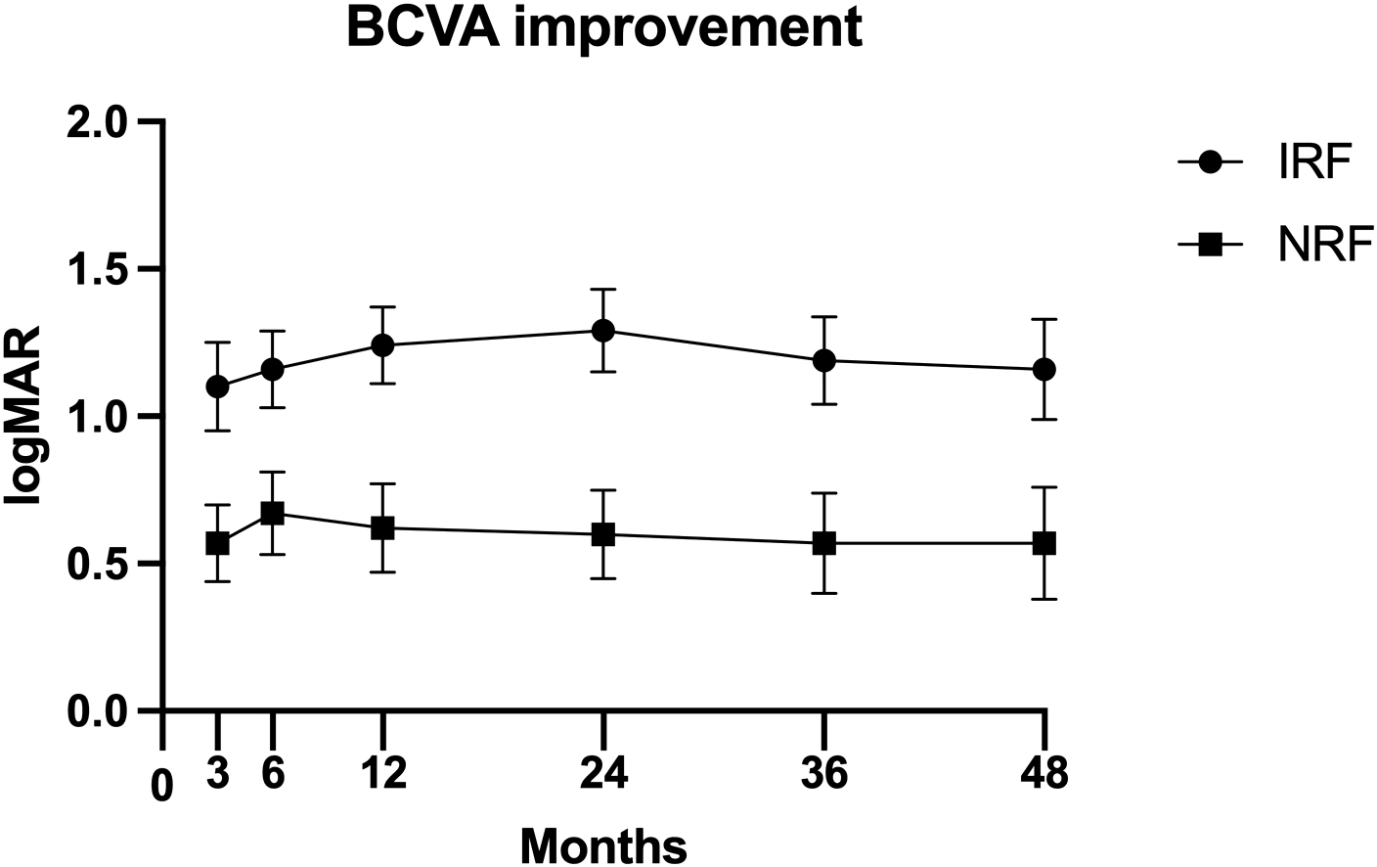
Postoperative best corrected visual acuity (BCVA) improvement [mean ± standard error of the mean (± SEM)] at 3 months, 6 months, 1 year, 2 years, 3 years, and 4 years after primary vitrectomy.

## 4 Discussion

As common microvascular complications of DM, DN and DR are reported to be closely related (20, 32). Despite the different functions of the kidney and the eye, these organs share similar molecular structure and developmental pathways, leading to substantial overlap in disease-causing risk factors, pathogenesis, and pathological changes between DR and DN (33). It has been reported that the severity of ocular damage in DR correlates with the severity of renal impairment in DN, and the two conditions exhibit reciprocal predictive effects (20). A cross-sectional study has demonstrated that DR, particularly PDR, serves as an independent predictor of DN (34). Additionally, subclinical DN can be predicted by eGFR in DR patients, even in the absence of proteinuria (35). However, as two different organs, the kidney and eye possess unique microenvironments and physiological processes, leading to different diabetic microangiopathic damage in the two. Furthermore, different susceptibility genes (36, 37) and involved cytokines have been found in the two diseases (38, 39). Additionally, DR is a neurovascular disease resulting from the destruction of the retinal neurovascular unit (40). Patients with DR do not necessarily have DN, and vice versa (41, 42). Thus, DR and DN demonstrate both parallel and non-parallel patterns in their disease onset and progression (17, 32).

In our study, 68.8% of PDR patients had DN, while 31.2% of PDR patients did not. Similarly, Agardh E et al. reported that 35% of 100 insulin-treated PDR patients showed no DN changes (43). Meanwhile, Bermejo S et al. conducted a multi-center and retrospective study in 832 diabetic patients with renal biopsy data. After removing missing data, they found that 65.6% of 221 DR patients had DN and 70.7% of 205 DN patients had DR (44). Additionally, Cao X et al. demonstrated that 64 of 98 (65.3%) Chinese patients with simple DN had DR (41). However, Dong Z at al. screened T2DM patients who underwent renal biopsy and found that DR prevalence was 82.3% in the DN group and 7.9% in the non-diabetic renal disease (NDRD) group, indicating that diabetic patients without DR were more likely to develop NDRD (42). The absence of DR in some DN patients may be due to two key factors: 1) renal biopsy was not performed in some clinically diagnosed DN patients, potentially misclassifying cases; 2) some NDRD (e.g., IgA nephropathy) are included in the DN group, which results in the underestimation of DR prevalence in DN. In addition, disparities in diagnostic means of DN and DR may also contribute. A study of 138 patients with insulin-dependent DM found that those without retinopathy rarely developed nephropathy, while retinopathy was common in those without nephropathy (45). This discrepancy may arise because the retina is more susceptible to diabetic damage than the kidney, and fundus examination, being less invasive than kidney biopsy, yields higher DR detection rates (46). However, most prior studies are retrospective with relatively small sample sizes. Large-scale prospective studies are needed in the future to clarify the association between these two diabetic microvascular complications.

Li J et al. reported a higher percentage of incomplete pan-retinal photocoagulation (64.8% vs. 42.3%), severe fibrovascular membrane (43.7% vs. 25.5%), macula-involved TRD (46.5% vs. 24.1%), and extensive retinal vascular closure (63.3% vs. 13.4%) in the IRF group. They found no significant difference in baseline corrected visual acuity (28). Our study demonstrated no significant difference in the percentage of preoperative retinal photocoagulation, fibrovascular membrane, and macular detachment, but worse baseline BCVA, in the IRF group. Additionally, the IRF group in our study had a lower percentage of rubeosis iridis (6.2% vs. 19.0%) than the NRF group. Similar to our results, Larrañaga-Fragoso P et al. reported worse baseline BCVA in the group with poor renal function (p = 0.039) (27). In contrast, Liu J et al. demonstrated that patients with renal dysfunction had better baseline BCVA (p = 0.006) (29). The discrepancies in these results may be due to different inclusion criteria, grouping methods, and sample sizes.

Our study showed no significant differences in intraoperative outcomes, including the rate of cataract surgery, intraoperative retinal photocoagulation, gas tamponade, silicone oil tamponade, intraoperative complications (retinal dialysis and/or iatrogenic retinal breaks), and MIVS application between the IRF and NRF groups. Similarly, Li et al. reported no significant differences in the rate of combined cataract surgery, the use of silicone oil tamponade, laser points, and operative time between the two groups. However, they recorded a higher percentage of severe intraoperative bleeding, intraocular subretinal fluid drainage, and perfluorocarbon liquids in the IRF group (28). Larrañaga-Fragoso P et al. detected no significant difference in the percentage of air and gas tamponade between groups categorized by renal function. Notably, they found that patients with poor renal function required less silicone. They postulated that it is because patients with poor renal function tend to present late with more fibrotic membranes (27). Additionally, the overall percentage of silicone tamponade in the study by Larrañaga-Fragoso P et al. was higher than ours (24.8% vs. 2.3%). This may be because all their participants had delamination or segmentation of pre-retinal membranes. Although 90 mL/min/1.73 m^2^ was used as the threshold for subgrouping, the study by Liu J et al. showed no significant differences between the IRF and NRF groups in terms of PPV time and the percentage of iatrogenic hole and tamponade (air, gas, and silicone oil) (all p > 0.05), which can be explained by the similar expression level of VEGF-A in the vitreous and aqueous humor and serum between the two groups (29).

Postoperative VH, NVG, and DME are common complications of PPV. Our study found no significant intergroup difference in the incidences of postoperative VH and/or RD, NVG, and DME. Similarly, Li J et al. demonstrated no significant difference in VH incidence (5.4% and 5.7%) and persistent DME (2.7% and 4.2%) between the IRF group and NRF groups. No cases of NVG developed during the 3-month follow-up period (28). However, Kameda Y et al. reported that the 6-month incidences of VH and NVG in the IRF group were significantly higher than those in the NRF group (43% vs. 10% and 17% vs. 2%, respectively). Additionally, patients with postoperative VH and NVG had lower eGFRs than those without (p < 0.001 and p = 0.007, respectively) (26). Larrañaga-Fragoso P et al. also showed that late VH occurred more frequently in patients with poor renal function (p = 0.003) (27). Liu J et al. reported a 60% overall DME rate at 3 months after surgery (50% in IRF and 68% in NRF) (29), which far exceeded our findings (8.6% in total, 9.3% in IRF, and 7.9% in NRF) and data reported by Li J et al. (28). The disparity may be due to their exclusion of patients who received preoperative anti-inflammatory therapy.

This study observed no significant between-group differences in postoperative BCVA at each follow-up point, consistent with previous studies’ results (27–29). Li J et al. reported no significant difference in the percentage of corrected visual acuity changes increased for more than two lines in patients with and without IRF (46.5% vs. 54.4%) (28). However, our data showed that the IRF group exhibited significantly greater BCVA improvement than the NRF group at all follow-up points. Similarly, Larrañaga-Fragoso P et al. demonstrated that patients with IRF achieved greater post-operative BCVA improvement than those with NRF at 6 months (27). In contrast, Liu J et al. found that the NRF group gained greater post-operative BCVA improvement at 3 months (29). The divergent findings across these studies may stem from the fact that the group with poorer baseline BCVA had greater room for visual improvement. Additionally, this study found no significant between-group differences in the proportion of postoperative BCVA ≥20/40 and ≥20/30 at both 2 and 4 years. Notably, Liu J et al. reported a lower proportion of patients in the IRF group with postoperative BCVA ≥ 6/12 at 3 months (6.7% vs. 13.3%), and Larrañaga-Fragoso P et al. similarly demonstrated a lower proportion of patients achieving BCVA ≥ 6/12 at 6 months in the IRF group (10.7% vs. 21.4%). This discrepancy may be attributed to the relatively short follow-up periods in the studies by Liu J et al. and Larrañaga-Fragoso P et al.

Our study is the first to investigate the association of renal function and long-term outcomes of PPV in patients with PDR. Patients in our cohort were followed up for at least 2 years and up to 4 years post-PPV. A comprehensive comparison was also conducted including baseline characteristics, intraoperative outcomes, postoperative complications, and postoperative visual outcomes. However, this study has several limitations. First, the retrospective design necessitates prospective studies to further confirm our findings. Second, all surgical procedures were performed by two surgeons, and subtle variations in surgical technique may affect anatomic and visual results. Third, although no statistical difference in the proportion of MIVS was observed between the two groups, the therapeutic effects of three-port 20-, 23-, and 25-G PPVs require further evaluation.

## 5 Conclusion

In conclusion, our secondary analysis demonstrated that renal insufficiency does not adversely affect the surgical outcomes of PPV for PDR. Thus, renal function alone should not be regarded as a prognostic indicator for PPV in patients with PDR.

## Data Availability

The original contributions presented in the study are included in the article/**Supplementary Material**. Further inquiries can be directed to the corresponding author.
